# F-18 Fluorodeoxyglucose Positron Emission Tomography/ Computed Tomography Findings of Isolated Gastric Tuberculosis mimicking Gastric Cancer and Lymphoma

**DOI:** 10.5005/jp-journals-10018-1270

**Published:** 2018-05-01

**Authors:** Remzi A Akdogan, A Akdogan Halil Rakici, Serkan Güngör, Recep Bedir, Elif Akdogan

**Affiliations:** 1 Department of Gastroenterology, Faculty of Medicine, Recep Tayyip Erdğan University, Rize, Turkey; 2 Department of Nuclear Medicine Research and Training Hospital, Recep Tayyip Erdogan University, Rize, Turkey; 3 Department of Pathology, Faculty of Medicine, Recep Tayyip Erdogan University, Rize, Turkey; 4 Department of Hematology, Faculty of Medicine, Recep Tayyip Erdogan University, Rize, Turkey

**Keywords:** Computed tomography, Isolated gastric tuberculosis, Positron emission tomography.

## Abstract

**Introduction:**

Tuberculosis (TB) infection is still a challenging health issue, especially in developing countries. Diagnosing extrapulmonary infections, especially isolated organ involvement, is difficult in most cases even with the radiological, endoscopic, and histopathological examinations done for accurate diagnosis. Here we describe a case of isolated gastric TB with specific F-18 fluorodeoxyglucose (FDG) positron emission tomography (PET)/computed tomography (CT) findings mimicking gastric cancer/ lymphoma.

**Case report:**

A 20-year-old male patient was admitted to our hospital with abdominal pain in the epigastric region, weight loss, and fever especially at nights for 2 months. Physical examination was normal. Hemoglobin was 9.6 gm/dL; the patient had iron deficiency anemia. Upper gastrointestinal (GI) endoscopy was suggestive of gastric ulcer mimicking malignancy. F-18 FDG PET/CT revealed multiple hypermetabolic malignant lymphadenopathies in the abdomen and diffuse gastric wall thickening as linitis plastica and multiple hypermetabolic peritoneal implants in the omentum. Exploratory laparotomy was done for tissue diagnosis and exploration of the peritoneum for TB infection, lymphoma, and Crohn’s disease to make differential diagnosis. Histopathology revealed granulomatous lymphadenitis with granulomas including giant cells, suspecting TB. Patient was put on antituberculosis treatment (ATT). After treatment, the complaints resolved, and he gained weight. Fusion PET/CT exhibited a complete response to ATT with no residual disease.

**Conclusion:**

According to our knowledge, this is the first report about F-18 FDG PET/CT findings in the diagnosis of isolated gastric TB. F-18 FDG PET/CT may provide help in the diagnosis and follow-up of isolated gastric TB in challenging cases.

**How to cite this article:** Akdogan RA, Rakici H, Güngör S, Bedir R, Akdogan E. F-18 Fluorodeoxyglucose Positron Emission Tomography/Computed Tomography Findings of Isolated Gastric Tuberculosis mimicking Gastric Cancer and Lymphoma. Euroasian J Hepato-Gastroenterol 2018;8(1):93-96.

## INTRODUCTION

The diagnosis of isolated gastric TB is mostly challenging and clinicians should make differential diagnosis between Crohn’s disease, gastric cancer, lymphoma, and TB in these cases. Isolated gastric TB is rare and its diagnosis is not easy even with the radiological, endoscopic, and histopathological examinations done for the accurate diagnosis. Here, we describe a case of isolated gastric TB with specific F-18 FDG PET/CT findings mimicking gastric cancer/lymphoma.

## CASE REPORT

**Figs 1A to D:**
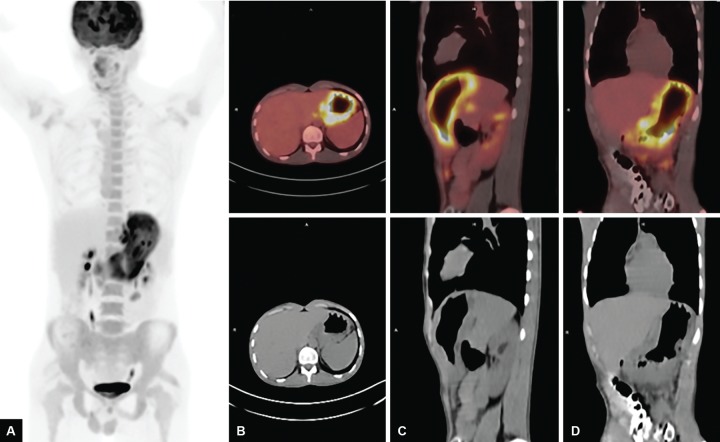
Maximum intensity projection (MIP), axial, sagittal, and coronal F-18 FDG PET/CT images before ATT; MIP image (A) demonstrated diffusely increased FDG uptake throughout the stomach. Axial (B), sagittal (C), and coronal (D) PET/ CT images showed intense FDG uptake in the thickened gastric wall, and intra-abdominal enlarged lymph nodes

**Fig. 2:**
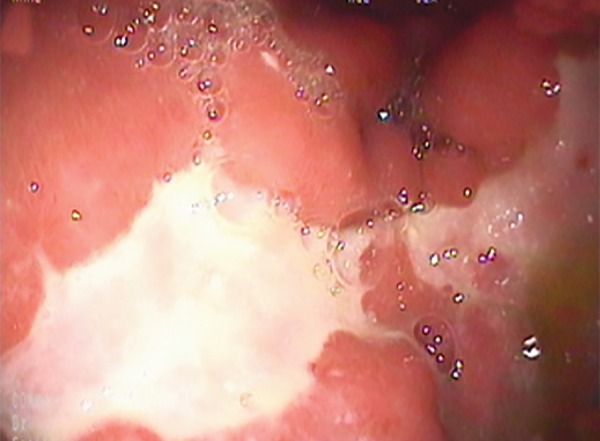
Gastric ulcer on incisura angularis

**Fig. 3:**
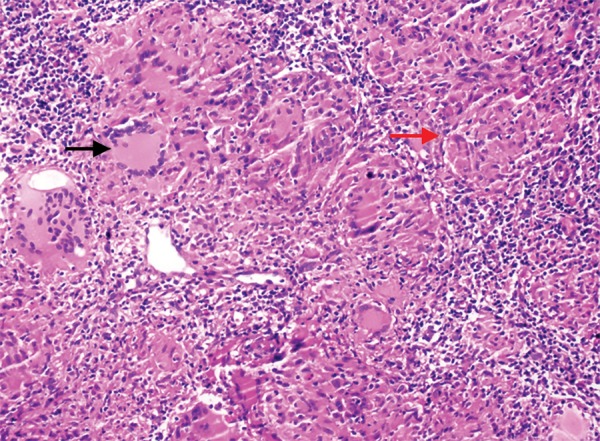
Langhans-type giant cells (black arrow), granulomas composed of epithelioid histiocytes (red arrow) (H&E 200*)

**Figs 4A to D:**
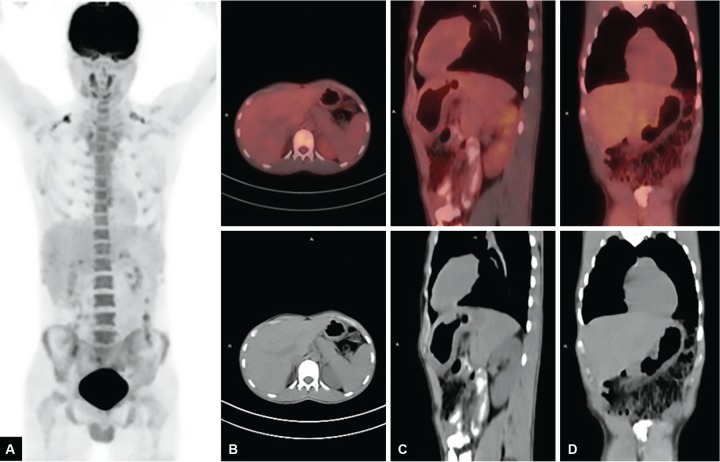
Follow-up F-18 FDG PET/CT images [maximum intensity projection (A), axial (B), sagittal (C), and coronal (D)] exhibited complete response to ATT with no residual disease

A 20-year-old male patient was admitted to our hospital with abdominal pain in the epigastric region, weight loss, and fever for 2 months. He had low back pain for 6 months and was evaluated for this complaint in another hospital. He had significant weight loss of 10 kg in 2 months and fever especially at nights. Past or family history revealed no signs of chronic or significant illnesses. Physical examination was normal. X-ray chest was normal. Anteroposterior plain radiograph of the sacroiliac joints revealed grade II bilateral sacroiliitis. Human leukocyte antigen B27 was positive. Liver and kidney function tests were normal. Lactic acid dehydrogenase was 229 U/L, upper of normal limits. Hemoglobin was 9.6 gm/dL; the patient had iron deficiency anemia. Serum angiotensin-converting enzyme level was 10.7 U/L (normal 8.0-52). His human immunodeficiency virus status was negative. F-18 FDG PET/ CT revealed multiple hypermetabolic malignant lymphadenopathies at gastrohepatic, gastrosplenic, celiac, superior mesenteric, peripancreatic and hepatobiliary region, and paragastric region in size as 18 × 15 mm (SUV_max_: 12.3) and diffuse gastric wall thickening as linitis plastica (SUV_max_: 13.3), multiple hypermetabolic peritoneal implants in the omentum (SUV_max_: 5.7) and peritoneum, and mild hypermetabolic suspected malignant lymph nodes at left supraclavicular region (Fig. 1). Gastric ulcer at incisura angularis was detected on upper GI endoscopy (Fig. 2). Endoscopic biopsies were repeated for histopathological and microbiological differential diagnosis. Histopathological examination showed granulomatous gastritis, Langhans-type giant cells, granulomas composed of epithelioid histiocytes, ulceration, and exudates in the two samples. Real-time TB-PCR were negative, Erlich-Ziehl-Neelsen staining bacteria were negative. Gastric fluid examination revealed Gram-positive cocci, Gram-positive bacillus, and no leukocytes. Exploratory laparotomy was done to sample the biggest sized lymph nodes for tissue diagnosis and explore the peritoneum for TB infection, lymphoma, and Crohn’s disease to make differential diagnosis. During laparotomy, the abdomen and peritoneum were intact and normal; two lymph nodes were extracted for histopathological and microbiologic diagnosis. Histopathology of the lymph nodes extracted by exploratory laparotomy revealed granulamatous lymphadenitis with granulomas including giant cells, mostly suspecting TB (Fig. 3). Ankylosing spondylitis was also diagnosed. Bath ankylosing spondylitis disease activity index score was 1.0, disease activity was low. Patient was put on ATT consisting of (2HREZ/7HR) regimen as isoniazid, rifampicin, ethambutol, and pyrazinamide at therapeutic doses for initial 2 months followed by rifampicin and isoniazide in the same doses for the last 7 months. At the 6th week of treatment, he gained weight about 6 kg and he was feeling healthy. Hemoglobin was 12.6 gm/dL. We performed follow-up F-18 FDG PET/ CT. The F-18 FDG PET/ CT images (maximum intensity projection, CT, and fusion PET/ CT) exhibited a complete response to ATT with no residual disease (Fig. 4).

We have taken written informed consent from the patient reported in this study.

## DISCUSSION

In the GI tract, abdominal TB can be seen in any part of the GI system. The most commonly seen clinical manifestation of abdominal TB is abdominal lymphadenopathy. The most commonly involved parts of the GI tract are the ileocecal region and ascending colon. Isolated gastric TB is so rare and its diagnosis is mostly challenging.^1^ The lesions may be in different forms like ulcerative, proliferative, or ulceroproliferative. The proliferative and ulceroproliferative forms of the disease may come in view like a mass mimicking GI tract cancer even on advanced imaging evaluations and may show intense uptake on F-18 FDG PET/CT imaging.^2^ In our case, we performed F-18 FDG PET/CT evaluation for the differential diagnosis suspecting gastric cancer or lymphoma. In daily practice, oncologic evaluation is mostly done for staging, detection of recurrence, or extension of malignant diseases, and F-18 FDG PET/CT imaging is not specific for diagnosis of cancer. Although F-18 FDG PET/ CT is not sensitive for granulomatous diseases like TB, it is helpful for the clinicians in the evaluation of suspected or known TB cases.^3^ In cases of active TB, they mostly exhibit a high degree of FDG uptake in different degrees due to the grade of inflammatory activity.^4^ F-18 FDG PET/CT evaluation is highly sensitive for inflammation and infection, but it has a poor sensitivity for these conditions. The combined use of F-18 FDG and C-11 acetate may help to differentiate inflammation from neoplasms because C-11 accumulates in tumors but not in the inflammatory lesions.^5^ Martinez et al^6^ evaluated F-18 FDG-PET/CT as a noninvasive method for early therapeutic response in TB. In this study, lower SUV_max_ at month 1 of treatment is mentioned as a marker of early improvement with ATT and helps the clinicians to confirm TB diagnosis. In our case, we performed a follow-up F-18 FDG-PET/CT at 10th month of therapy to confirm our diagnosis and evaluate the success of ATT. Follow-up F-18 FDG PET/ CT exhibited a complete response to ATT with no residual disease. In the literature, there are quite enough reports about F-18 FDG PET/CT findings of abdominal TB and radiological findings of isolated gastric TB but there is no report about F-18 FDG PET/ CT findings of isolated gastric TB.^7^ According to our knowledge, our case report should be the first report about F-18 FDG PET/CT findings in the diagnosis of isolated gastric TB. F-18 FDG PET/CT may provide help in the diagnosis and follow-up of isolated gastric TB in challenging cases.
